# Supplementary material for: Uncovering the genetic basis of fruit volatiles in Fragaria vesca through GWAS reveals FvJMT2 as a methyl benzoate biosynthesis gene with bioactivity against Drosophila suzukii
Source: Hortic Res. 2026 Apr 16;13(9):uhag148. doi: 10.1093/hr/uhag148 (PMC13503962; doi:10.1093/hr/uhag148)
Supplement: Supplementary file 1 [file web_material_uhag148.zip › Supplementary Figures_Jimenez-Munoz_Urrutia_revised.pdf]

Supplemental Figures

A γ-decalactone

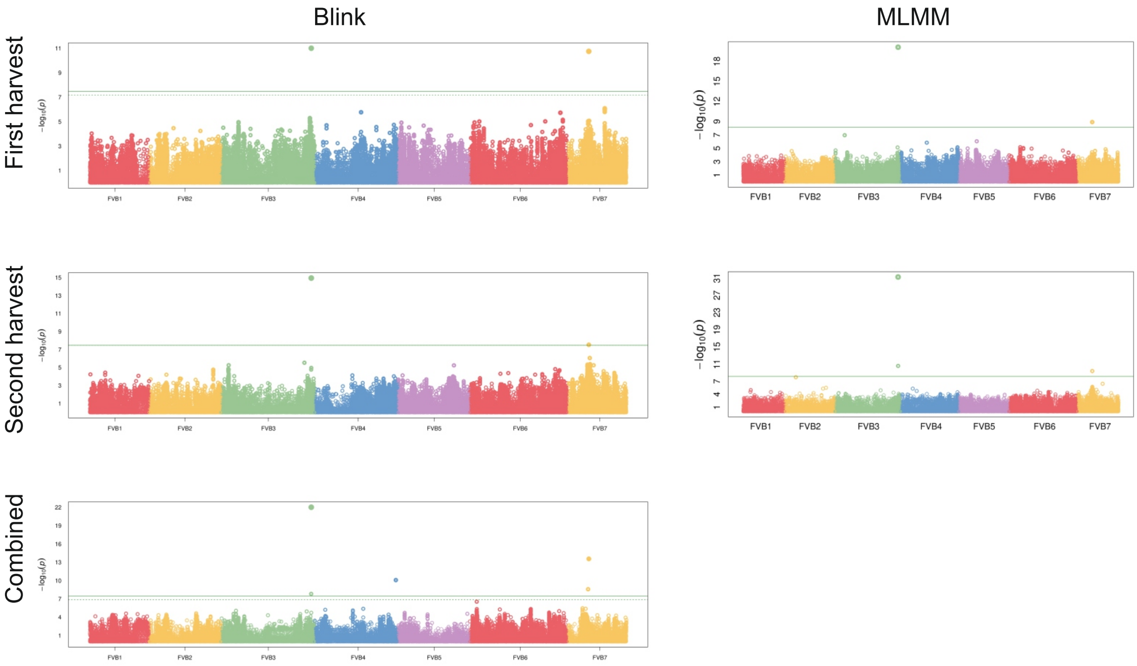

B Eugenol

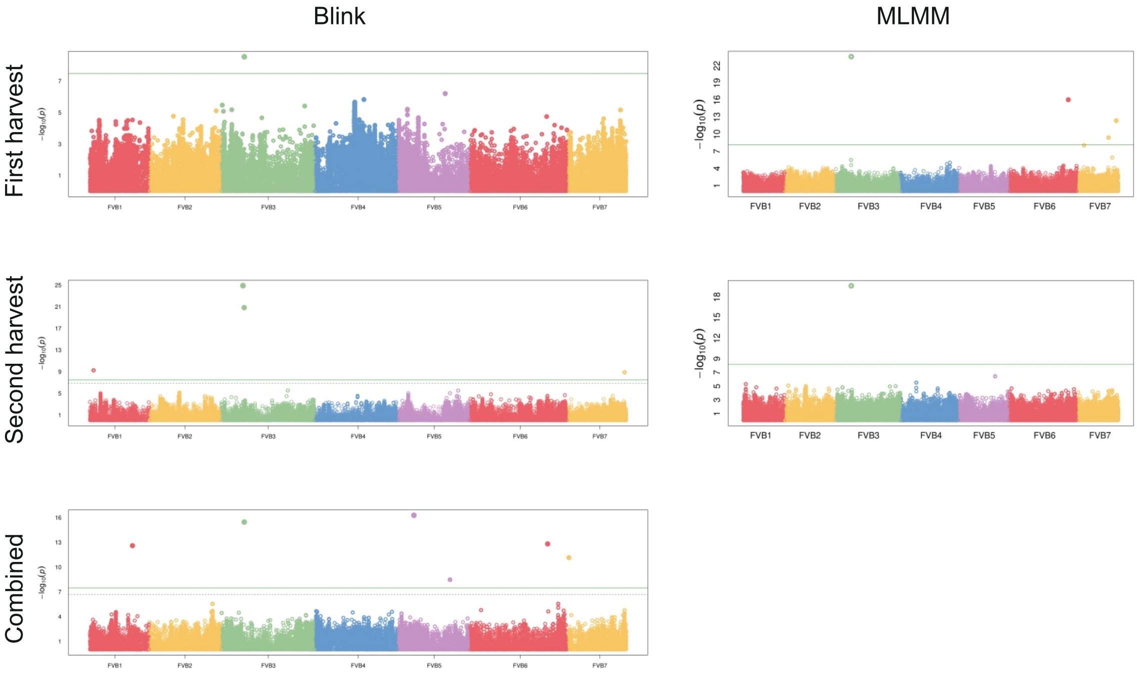

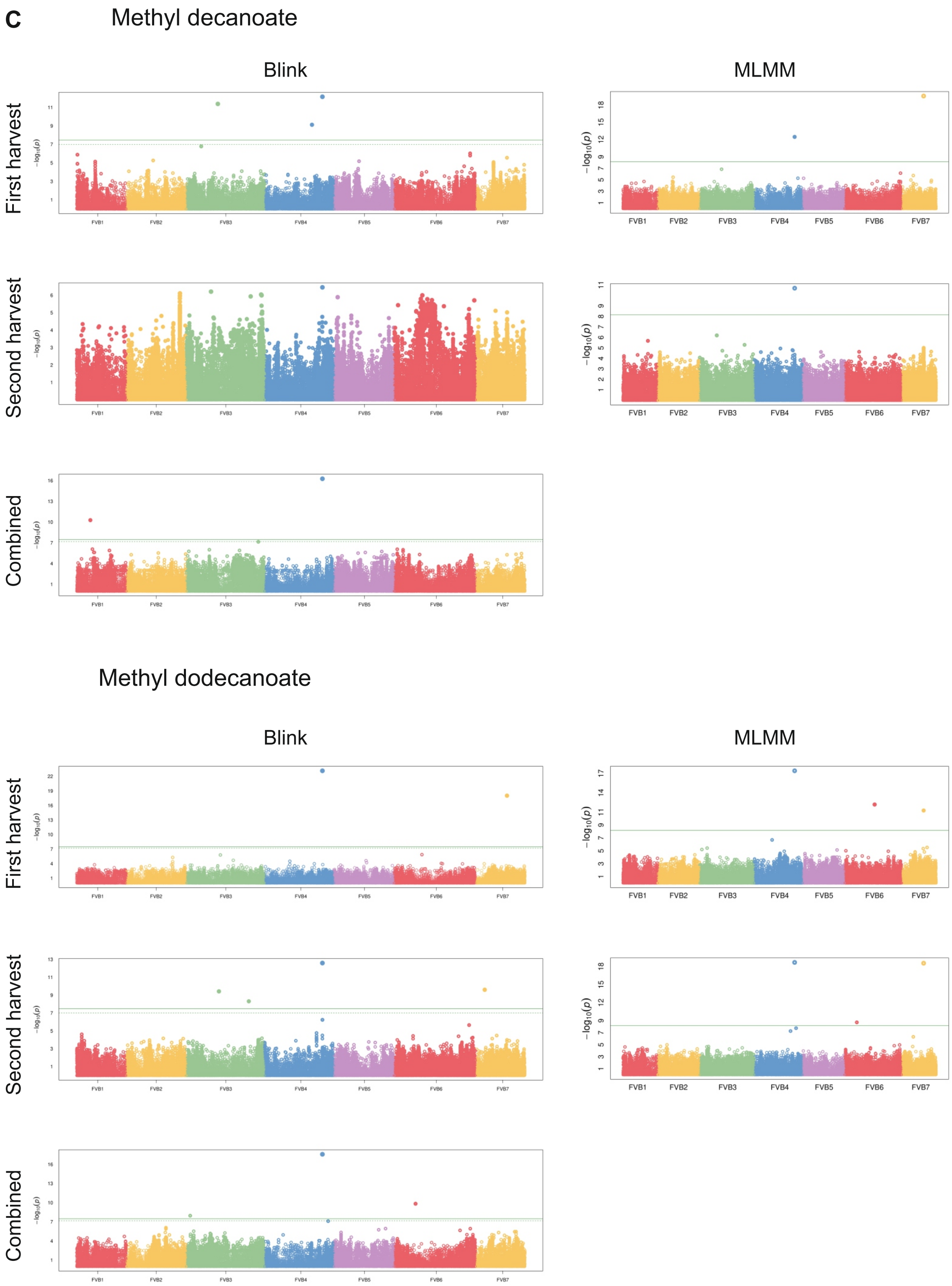
C
Methyl decanoate
Blink
MLMM
First harvest
Second harvest
Combined
Methyl dodecanoate
Blink
MLMM
First harvest
Second harvest
Combined
−log10(p)
FVB1
FVB2
FVB3
FVB4
FVB5
FVB6
FVB7

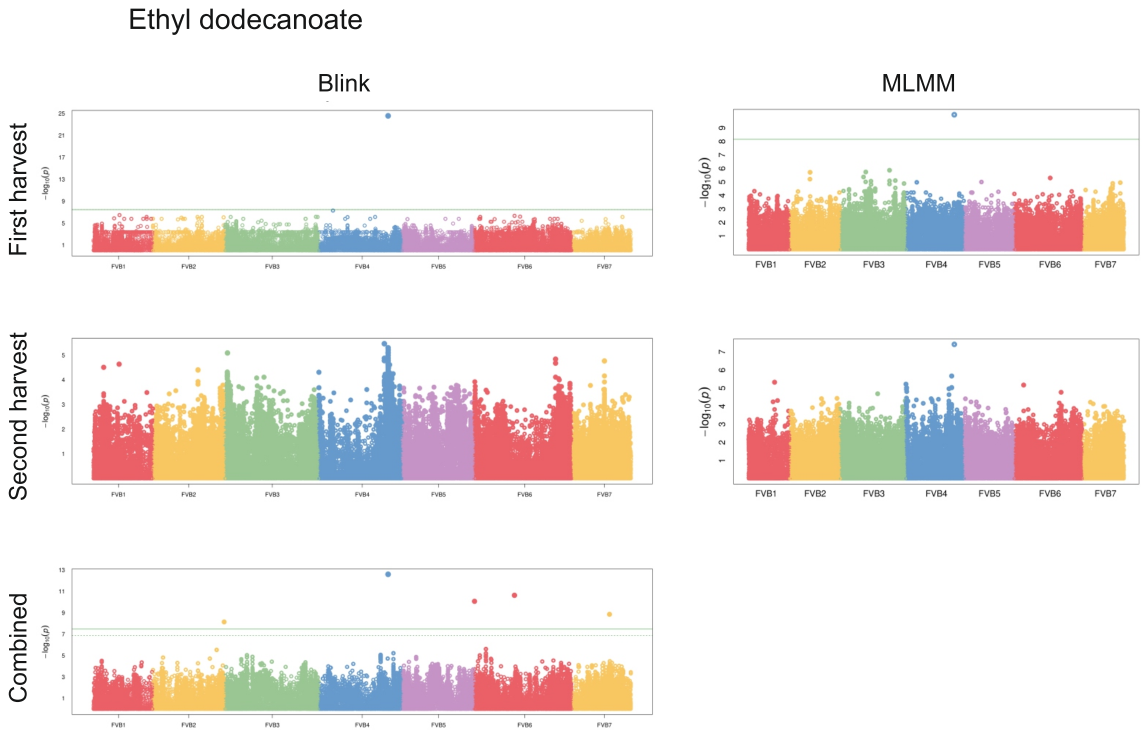
Ethyl dodecanoate
Blink
MLMM
First harvest
Second harvest
Combined
−log10(p)
FVB1
FVB2
FVB3
FVB4
FVB5
FVB6
FVB7

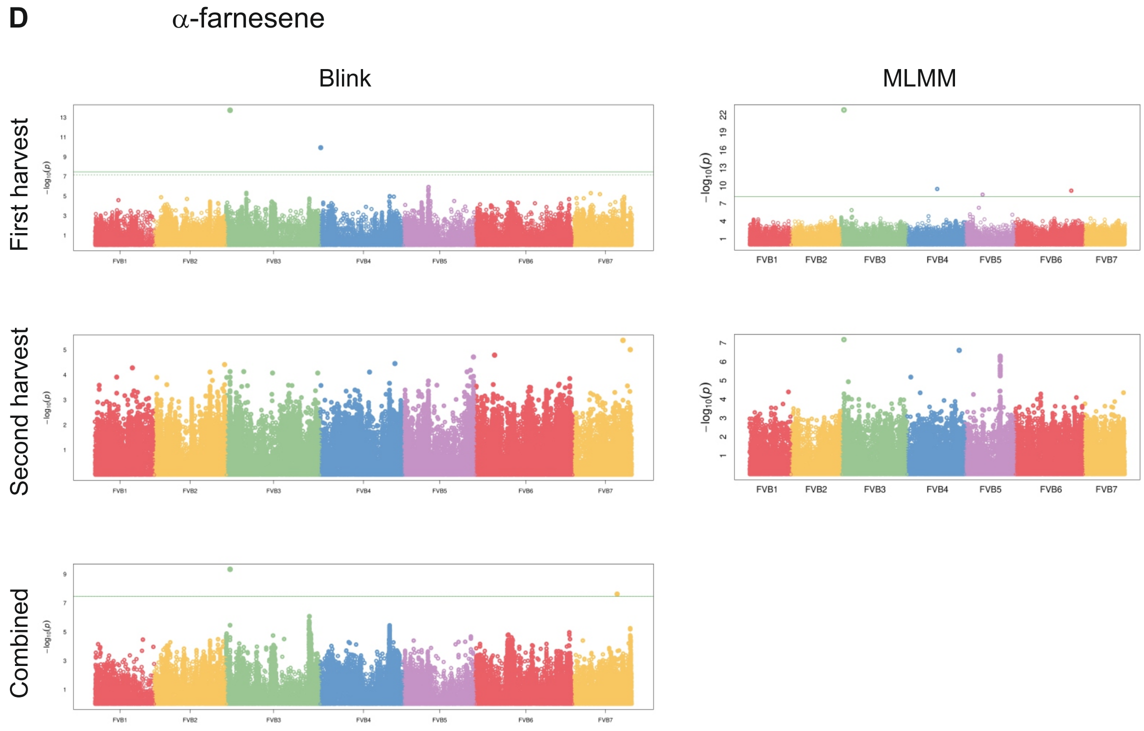
D
α-farnesene
Blink
MLMM
First harvest
Second harvest
Combined
−log10(p)
FVB1
FVB2
FVB3
FVB4
FVB5
FVB6
FVB7

E (*E*)-2-hexenal

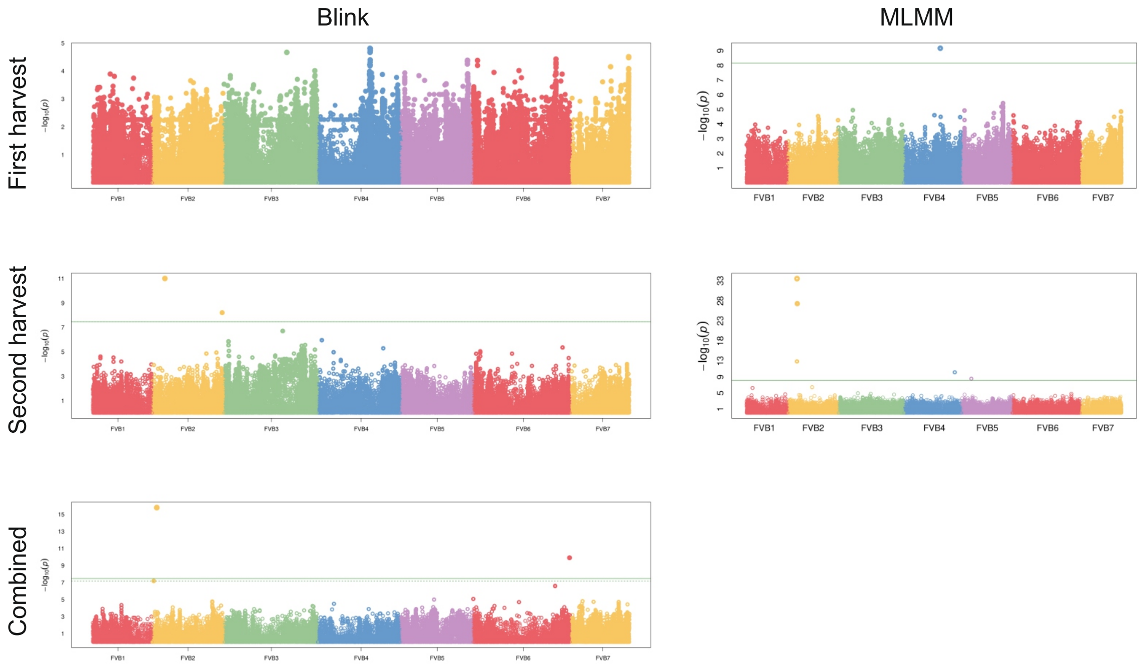

F α-pinene

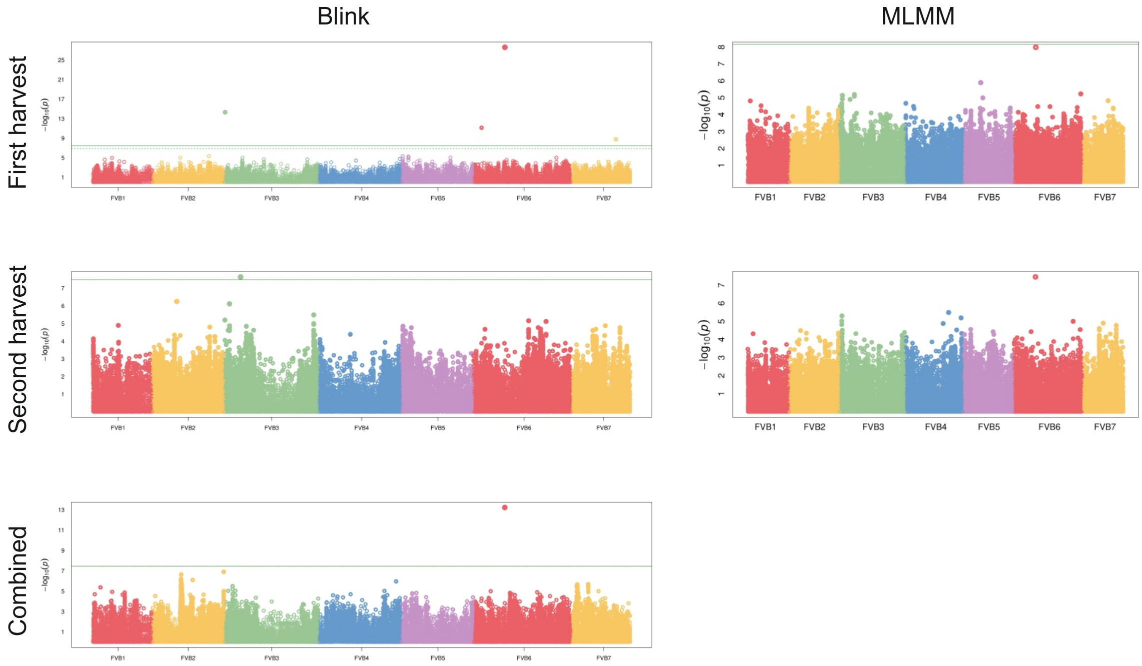

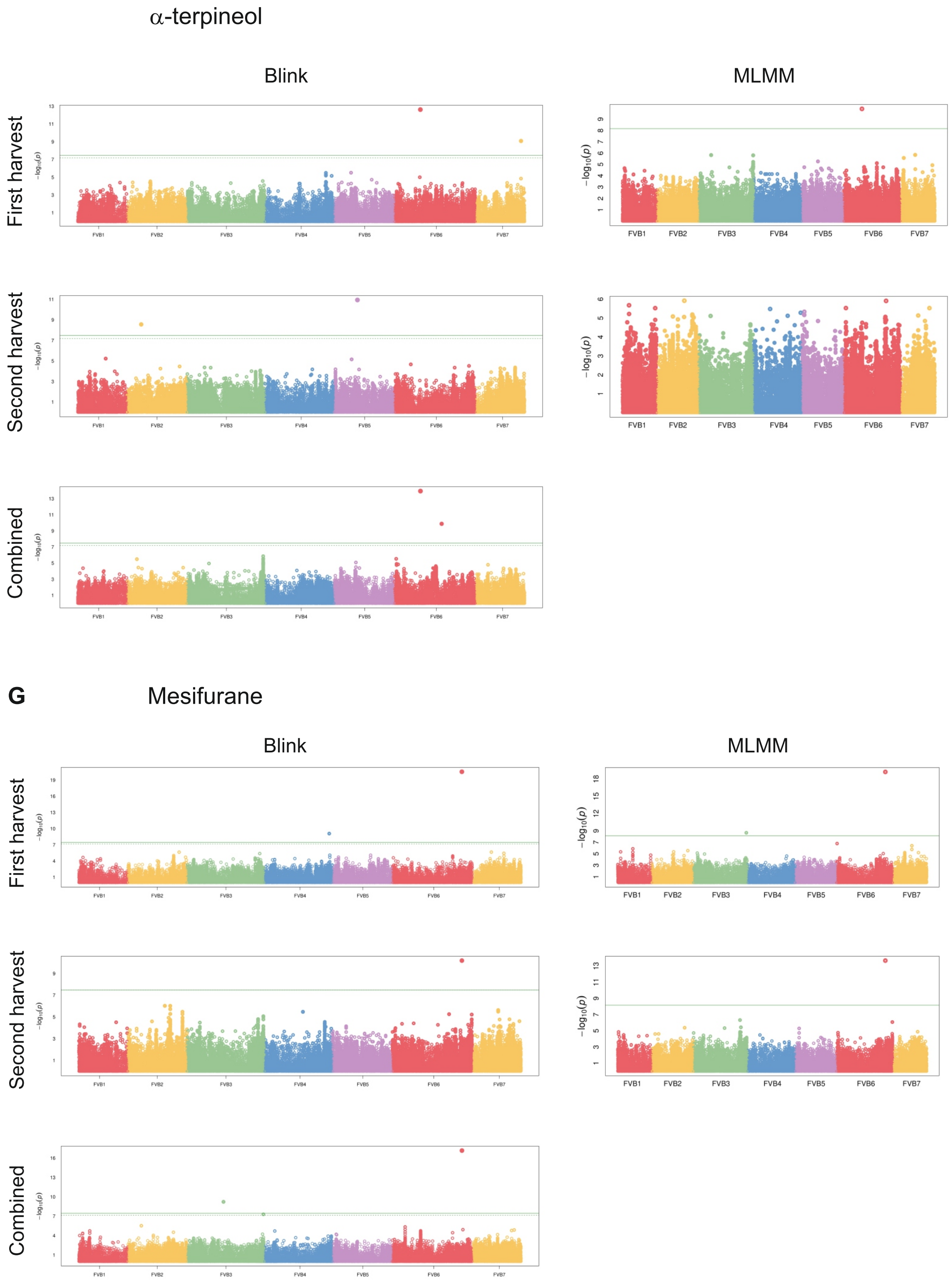
α-terpineol
Blink
MLMM
First harvest
Second harvest
Combined
G
Mesifurane
Blink
MLMM
First harvest
Second harvest
Combined
−log10(p)
FVB1
FVB2
FVB3
FVB4
FVB5
FVB6
FVB7

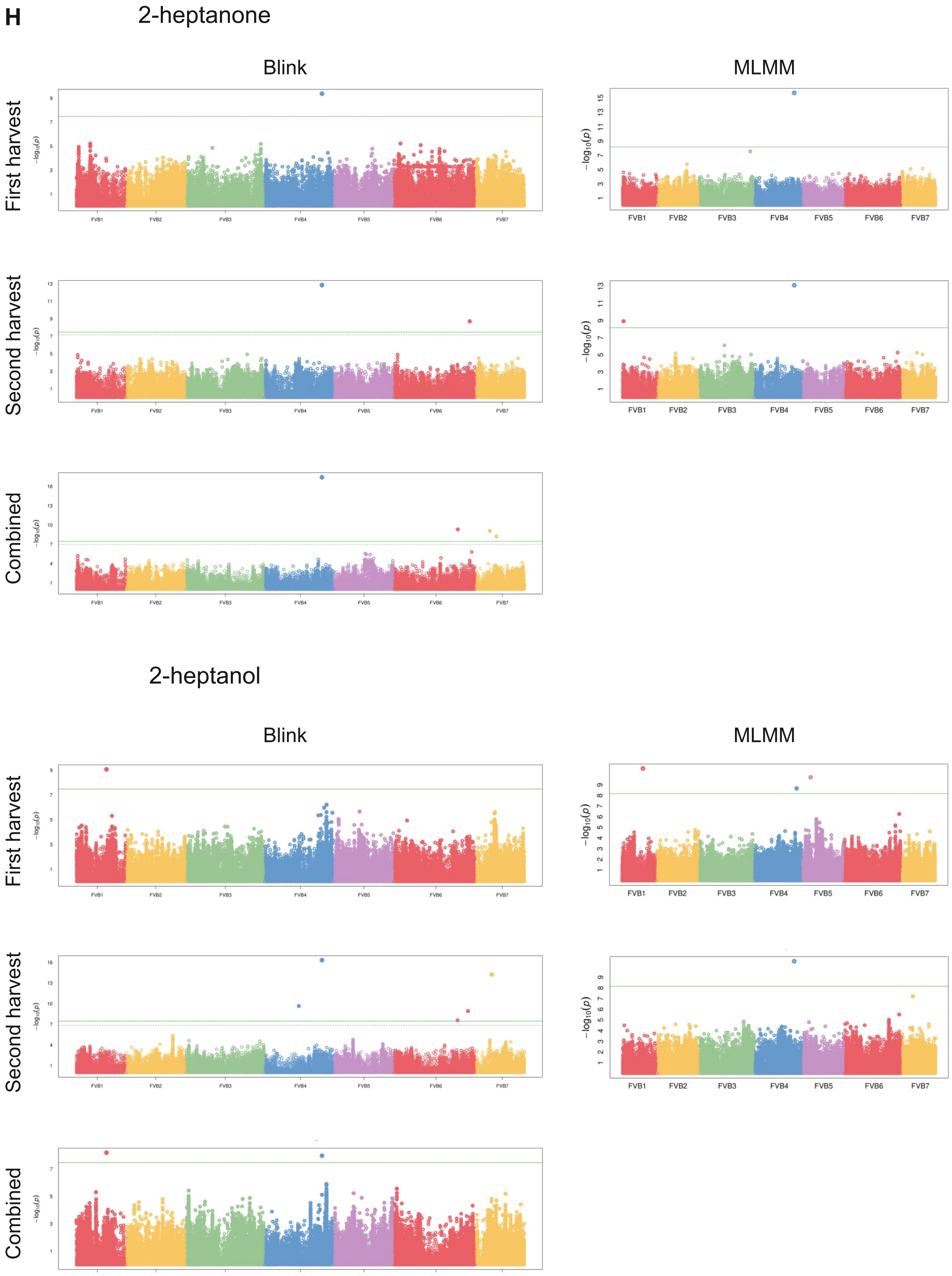
H
2-heptanone
Blink
MLMM
First harvest
Second harvest
Combined
−log10(p)
FVB1
FVB2
FVB3
FVB4
FVB5
FVB6
FVB7
2-heptanol
Blink
MLMM
First harvest
Second harvest
Combined

2-nonanone

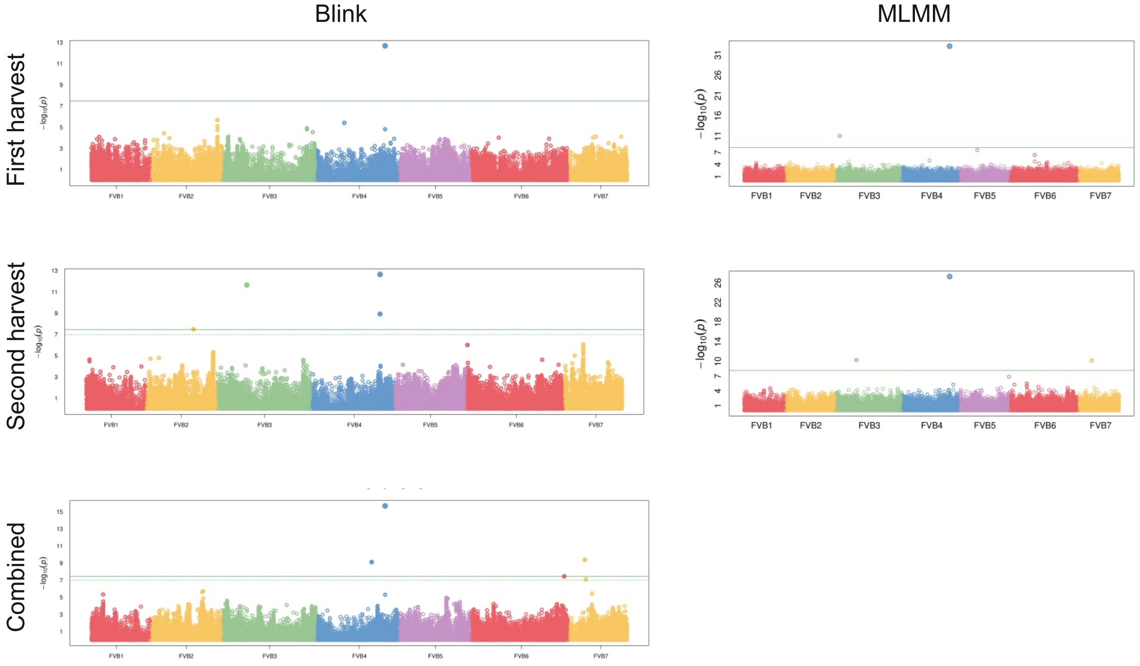

2-nonanol

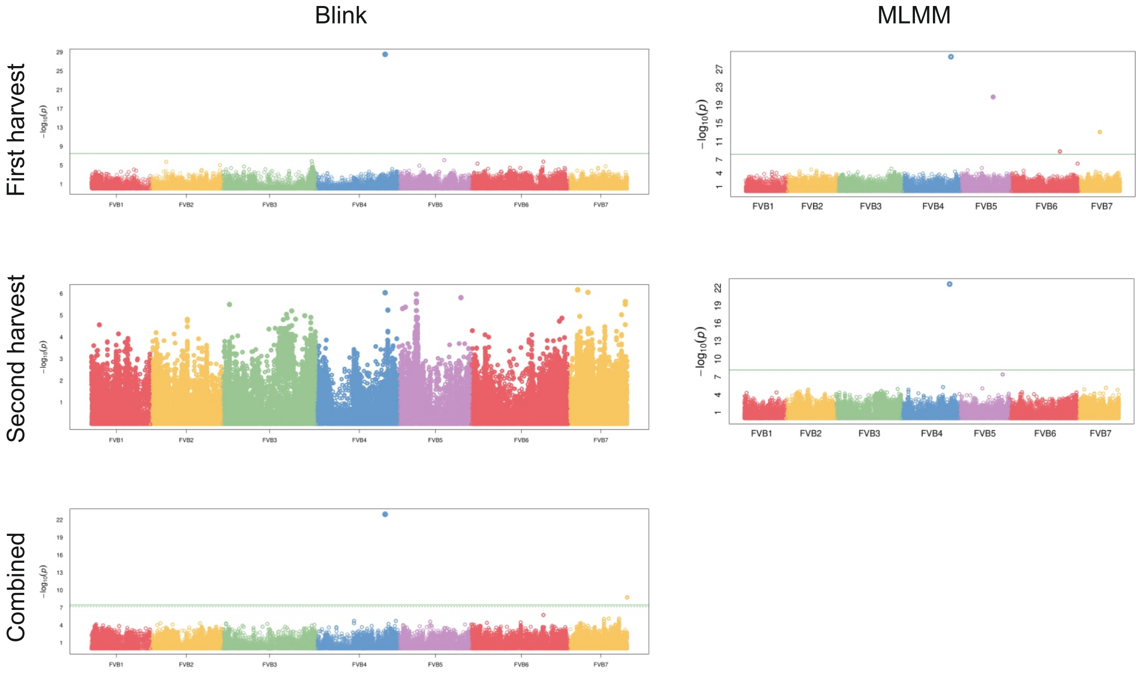

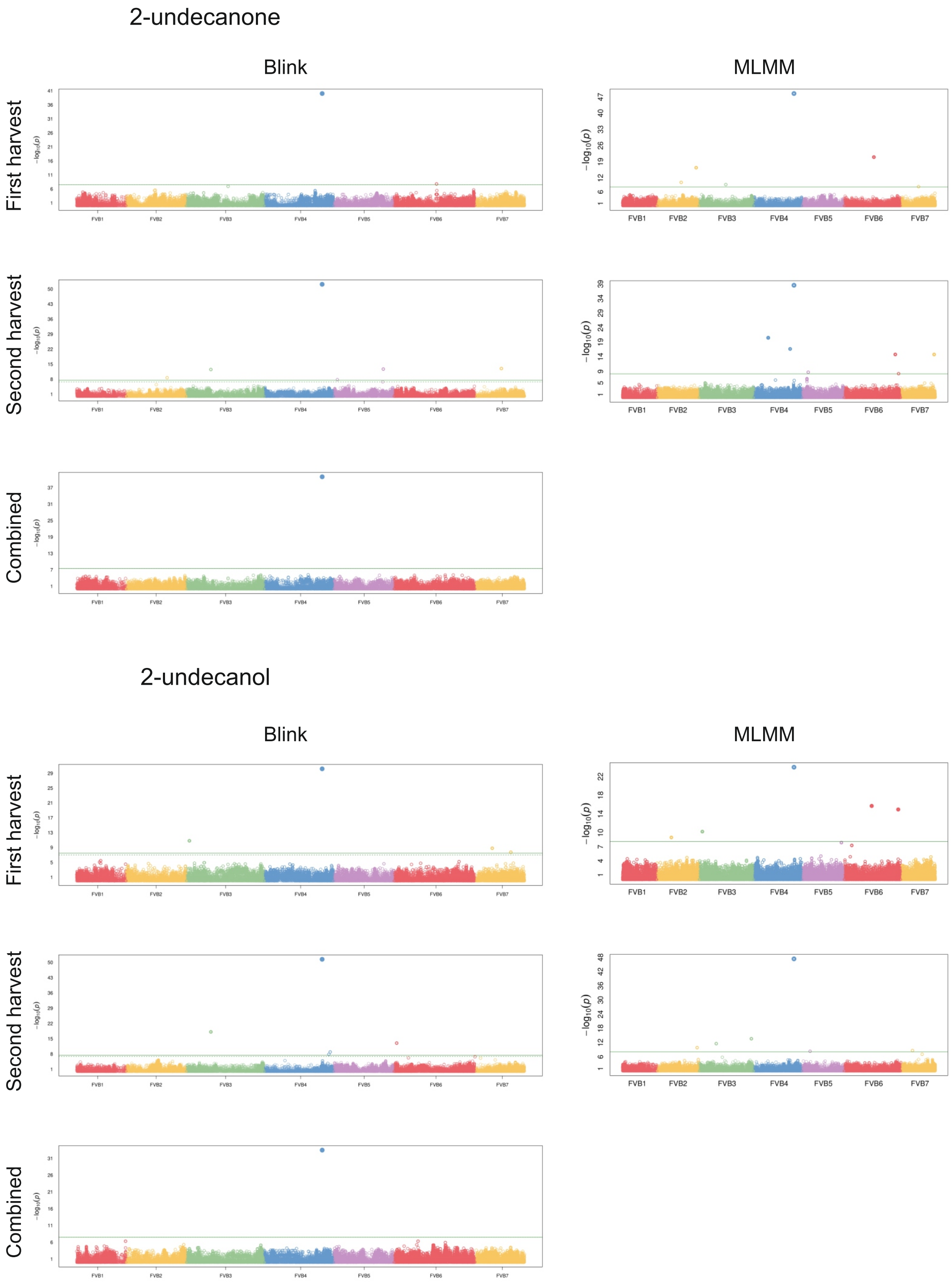

2-undecanone
Blink
MLMM
First harvest
Second harvest
Combined
2-undecanol
Blink
MLMM
First harvest
Second harvest
Combined
−log10(p)
FVB1
FVB2
FVB3
FVB4
FVB5
FVB6
FVB7

1-methyloctyl butanoate

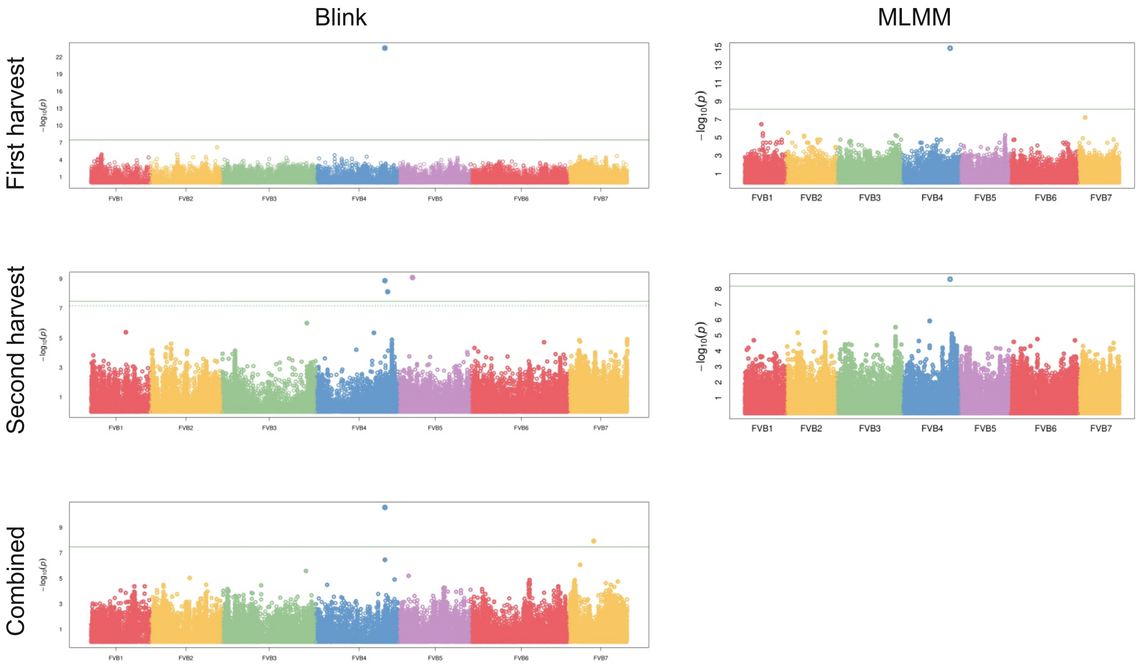

I Propyl butanoate

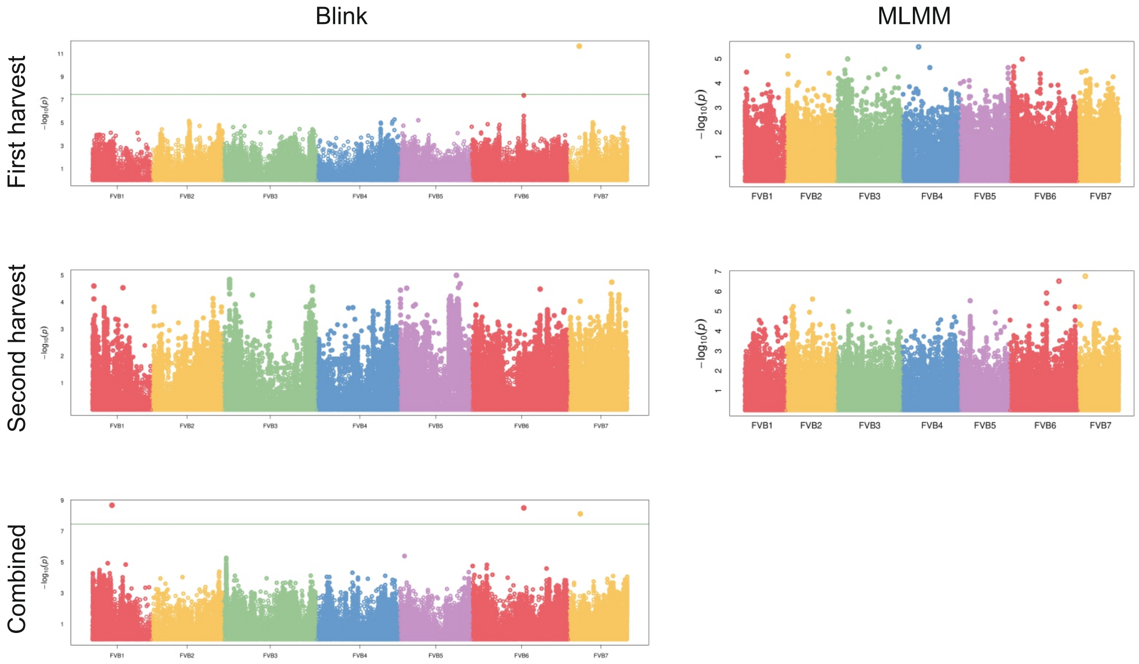

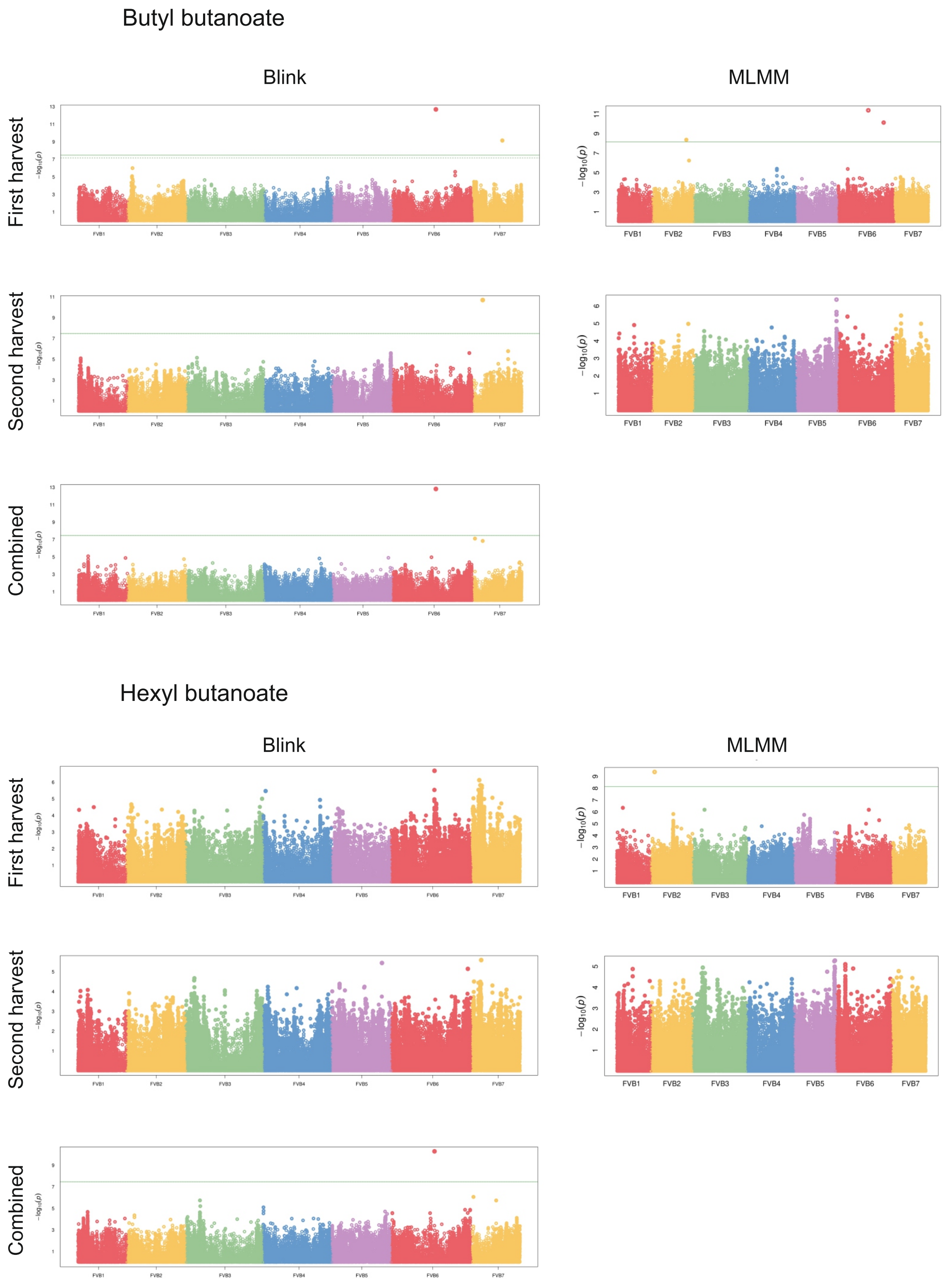
Butyl butanoate
Blink
MLMM
First harvest
Second harvest
Combined
Hexyl butanoate
Blink
MLMM
First harvest
Second harvest
Combined
−log10(p)
FVB1
FVB2
FVB3
FVB4
FVB5
FVB6
FVB7

J

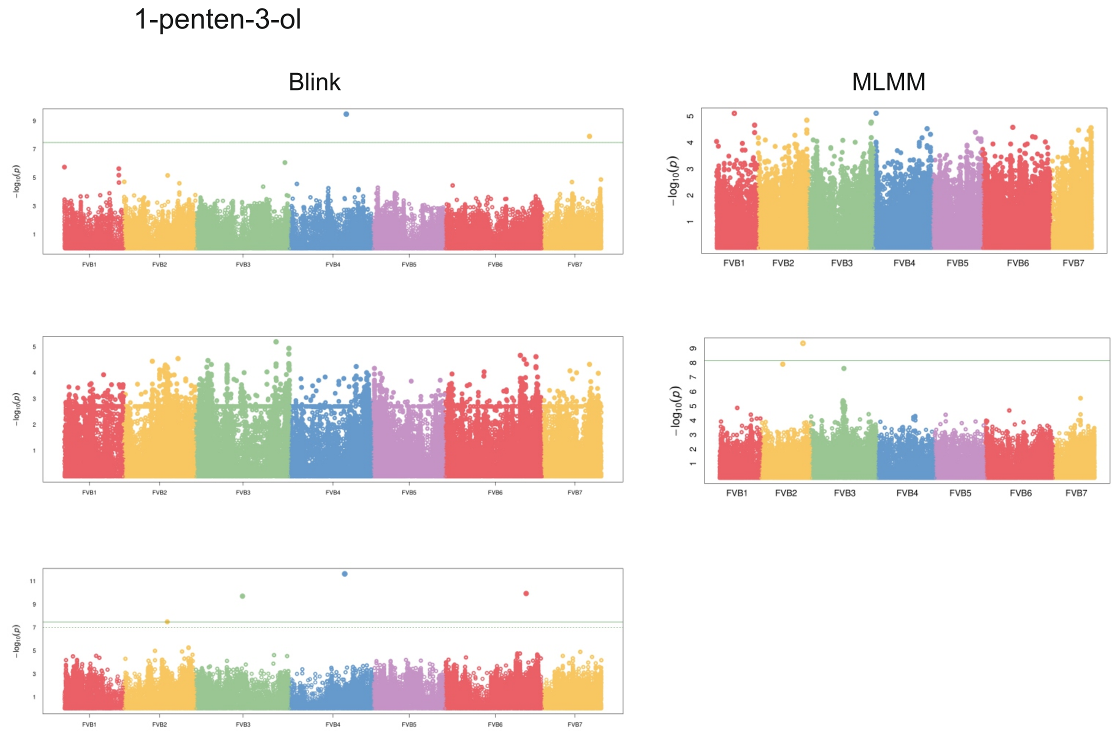
1-penten-3-ol
Blink
MLMM
First harvest
Second harvest
Combined
−log10(p)
FVB1
FVB2
FVB3
FVB4
FVB5
FVB6
FVB7

K

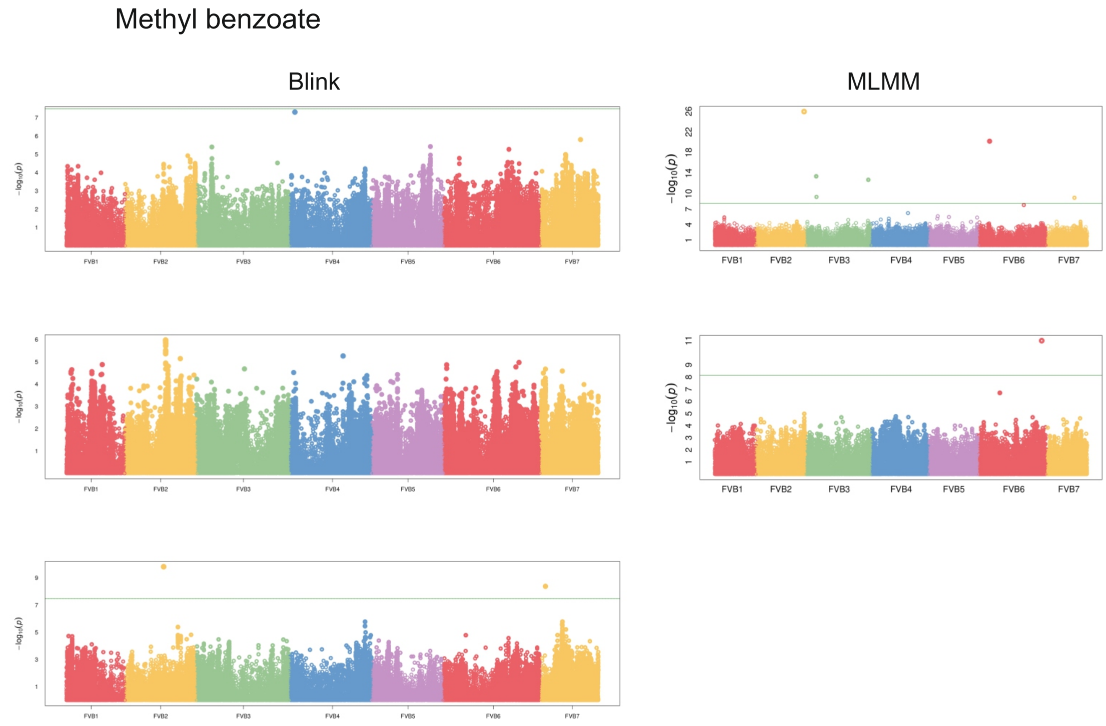
Methyl benzoate
Blink
MLMM
First harvest
Second harvest
Combined
−log10(p)
FVB1
FVB2
FVB3
FVB4
FVB5
FVB6
FVB7

Methyl cinnamate

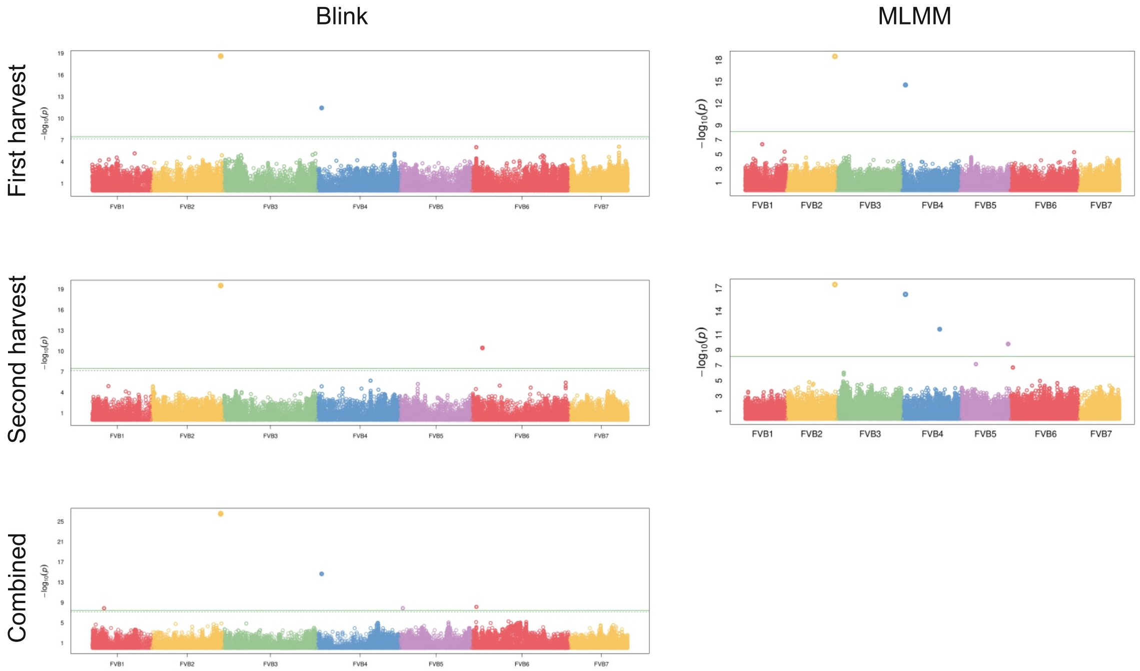

Figure S1. Manhattan plots of volatile compounds with stable GWAS associations. GWAS were conducted using the BLINK model for the first harvest, second harvest, and combined dataset (estimated marginal means), and the MLMM model for the first and second harvests only. The plots display volatile compounds for which consistent associations were detected in at least two datasets (individual harvests and/or combined dataset). Significant associations are shown for: γ-decalactone (A), eugenol (B), methyl decanoate, methyl dodecanoate and ethyl dodecanoate (C), (*E*,*E*)-α-farnesene (D), (*E*)-2-hexenal (E), α-pinene and α-terpineol (F), mesifurane (G), 2-heptanone, 2-heptanol, 2-nonanone, 2-nonaol, 2-undecanone, 2-undecanol and 1-methyloctyl butanoate (H), propyl butanoate, butyl butanoate and hexyl butanoate (I), 1-penten-3-ol (J), and methyl benzoate and methyl cinnamate (K).

```
                     M  E  L  M  Q  I  L  H  M  N  K  G  N  G  E  A  S  Y  A  Q
FvJMT2(C275)        ATGGAGCTAATGCAAATACTTCACATGAACAAAGGCAACGGCGAGGCTAGTTATGCCCAA 60
FvJMT2(A275)        ATGGAGCTAATGCAAATACTTCACATGAACAAAGGCAACGGCGAGGCTAGTTATGCCCAA 60
                     M  E  L  M  Q  I  L  H  M  N  K  G  N  G  E  A  S  Y  A  Q

                     N  S  K  V  Q  N  K  I  L  T  I  A  K  P  I  I  E  E  A  L
FvJMT2(C275)        AACTCCAAAGTTCAGAACAAGATATTAACAATCGCAAAGCCAATCATCGAAGAAGCCTTG 120
FvJMT2(A275)        AACTCCAAAGTTCAGAACAAGATATTAACAATCGCAAAGCCAATCATCGAAGAAGCCTTG 120
                     N  S  K  V  Q  N  K  I  L  T  I  A  K  P  I  I  E  E  A  L

                     L  K  L  L  G  S  N  N  N  D  M  A  L  E  S  M  G  I  A  D
FvJMT2(C275)        CTCAAACTGTTGGGGTCAAACAACAATGATATGGCATTGGAGAGTATGGGCATAGCGGAT 180
FvJMT2(A275)        CTCAAACTGTTGGGGTCAAACAACAATGATATGGCATTGGAGAGTATGGGCATAGCGGAT 180
                     L  K  L  L  G  S  N  N  N  D  M  A  L  E  S  M  G  I  A  D

                     L  G  C  S  S  G  P  N  T  F  L  L  I  S  E  I  M  D  V  I
FvJMT2(C275)        TTGGGTTGCTCATCTGGACCTAACACCTTTCTTCTCATATCGGAGATCATGGATGTCATA 240
FvJMT2(A275)        TTGGGTTGCTCATCTGGACCTAACACCTTTCTTCTCATATCGGAGATCATGGATGTCATA 240
                     L  G  C  S  S  G  P  N  T  F  L  L  I  S  E  I  M  D  V  I

                     R  A  K  L  S  S  G  L  L  H  P  S  S  S  S  M  E  F  R  V
FvJMT2(C275)        CGTGCCAAACTCAGCAGCGGCCTGCTGCATCCCTCGTCGTCGTCAATGGAGTTTAGAGTG 300
FvJMT2(A275)        CGTGCCAAACTCAGCAGCGGCCTGCTGCATCCCTAGTCGTCGTCAATGGAGTTTAGAGTG 300
                     R  A  K  L  S  S  G  L  L  H  P  *

                     F  L  N  D  L  F  S  N  D  F  N  T  V  F  M  S  L  P  A  F
FvJMT2(C275)        TTTCTCAACGACCTATTCAGCAACGACTTCAACACCGTCTTCATGTCACTTCCAGCATTC 360
FvJMT2(A275)        TTTCTCAACGACCTATTCAGCAACGACTTCAACACCGTCTTCATGTCACTTCCAGCATTC 360

                     H  N  K  L  R  K  D  D  R  Y  K  R  L  F  G  A  N  P  N  L
FvJMT2(C275)        CACAACAAGCTGAGAAAAGACGATCGCTACAAGAGACTGTTCGGGGCTAACCCCAACCTC 420
FvJMT2(A275)        CACAACAAGCTGAGAAAAGACGATCGCTACAAGAGACTGTTCGGGGCTAACCCCAACCTC 420

                     F  I  S  A  V  P  G  T  F  Y  G  R  L  F  P  R  N  S  L  H
FvJMT2(C275)        TTCATTTCCGCGGTGCCGGGAACTTTCTACGGCAGATTGTTTCCCAGAAACAGCCTTCAT 480
FvJMT2(A275)        TTCATTTCCGCGGTGCCGGGAACTTTCTACGGCAGATTGTTTCCCAGAAACAGCCTTCAT 480

                     F  V  H  S  S  S  S  L  H  W  L  S  Q  V  P  P  S  L  D  S
FvJMT2(C275)        TTCGTTCACTCGTCCTCCAGTCTTCACTGGCTGTCTCAAGTTCCCCCGAGCCTGGACAGC 540
FvJMT2(A275)        TTCGTTCACTCGTCCTCCAGTCTTCACTGGCTGTCTCAAGTTCCCCCGAGCCTGGACAGC 540

                     T  A  G  T  A  L  N  K  G  K  I  Y  I  S  K  S  S  P  Q  Y
FvJMT2(C275)        ACCGCCGGCACGGCCTTGAACAAGGGGAAGATCTACATTTCTAAGAGCAGCCCGCAATAT 600
FvJMT2(A275)        ACCGCCGGCACGGCCTTGAACAAGGGGAAGATCTACATTTCTAAGAGCAGCCCGCAATAT 600

                     V  L  D  A  Y  S  L  Q  F  H  K  D  F  S  M  F  L  K  S  R
FvJMT2(C275)        GTGTTGGATGCGTACTCACTTCAATTCCACAAGGACTTCTCAATGTTTCTCAAGTCTCGG 660
FvJMT2(A275)        GTGTTGGATGCGTACTCACTTCAATTCCACAAGGACTTCTCAATGTTTCTCAAGTCTCGG 660

                     A  E  E  I  V  G  G  G  R  M  V  L  S  F  I  G  R  P  T  L
FvJMT2(C275)        GCAGAAGAAATAGTTGGAGGAGGAAGAATGGTATTGTCATTCATAGGCAGGCCAACACTT 720
FvJMT2(A275)        GCAGAAGAAATAGTTGGAGGAGGAAGAATGGTATTGTCATTCATAGGCAGGCCAACACTT 720

                     D  P  T  T  E  Q  S  C  Y  H  W  E  L  L  A  L  A  L  M  S
FvJMT2(C275)        GACCCTACAACCGAACAGAGTTGCTACCATTGGGAGCTATTAGCCCTTGCATTGATGAGC 780
FvJMT2(A275)        GACCCTACAACCGAACAGAGTTGCTACCATTGGGAGCTATTAGCCCTTGCATTGATGAGC 780

                     M  V  S  D  G  L  V  E  E  E  K  V  D  S  F  N  A  P  Y  Y
FvJMT2(C275)        ATGGTTTCAGATGGTCTTGTGGAAGAGGAGAAAGTTGATTCCTTCAACGCTCCTTACTAT 840
FvJMT2(A275)        ATGGTTTCAGATGGTCTTGTGGAAGAGGAGAAAGTTGATTCCTTCAACGCTCCTTACTAT 840

                     A  P  C  A  E  E  L  K  S  E  L  Q  K  D  G  S  F  I  M  D
FvJMT2(C275)        GCTCCATGCGCAGAGGAACTGAAGTCGGAGCTGCAGAAGGACGGGTCCTTCATAATGGAC 900
FvJMT2(A275)        GCTCCATGCGCAGAGGAACTGAAGTCGGAGCTGCAGAAGGACGGGTCCTTCATAATGGAC 900
```

```
                   R  L  E  A  F  E  I  D  W  D  G  G  A  D  D  Q  A  I  T  M
FvJMT2(C275)      CGACTTGAAGCGTTCGAAATCGATTGGGATGGTGGTGCTGATGATCAAGCTATTACCATG 960
FvJMT2(A275)      CGACTTGAAGCGTTCGAAATCGATTGGGATGGTGGTGCTGATGATCAAGCTATTACCATG 960

                   N  G  F  D  H  D  Q  M  V  A  R  S  G  K  R  V  A  N  T  I
FvJMT2(C275)      AATGGTTTTGATCATGATCAAATGGTGGCTAGGAGTGGGAAGAGGGTGGCCAATACCATA 1020
FvJMT2(A275)      AATGGTTTTGATCATGATCAAATGGTGGCTAGGAGTGGGAAGAGGGTGGCCAATACCATA 1020

                   R  A  V  A  E  S  M  I  E  A  H  F  G  I  E  I  M  D  D  L
FvJMT2(C275)      AGAGCGGTGGCGGAGTCAATGATCGAAGCTCATTTTGGGATAGAGATTATGGATGATCTG 1080
FvJMT2(A275)      AGAGCGGTGGCGGAGTCAATGATCGAAGCTCATTTTGGGATAGAGATTATGGATGATCTG 1080

                   F  R  R  Y  A  E  L  V  T  K  H  L  L  E  T  R  T  K  Y  I
FvJMT2(C275)      TTTAGGAGGTACGCCGAACTCGTCACCAAACACTTGTTAGAGACAAGAACCAAGTATATC 1140
FvJMT2(A275)      TTTAGGAGGTACGCCGAACTCGTCACCAAACACTTGTTAGAGACAAGAACCAAGTATATC 1140

                   N  L  V  I  S  L  I  R  R  Y  *
FvJMT2(C275)      AATTTGGTCATTTCACTGATCAGAAGATATTGA       1173
FvJMT2(A275)      AATTTGGTCATTTCACTGATCAGAAGATATTGA       1173
```

Figure S2. Alignment of FvJMT2 CDSs and protein sequences of the two alleles.

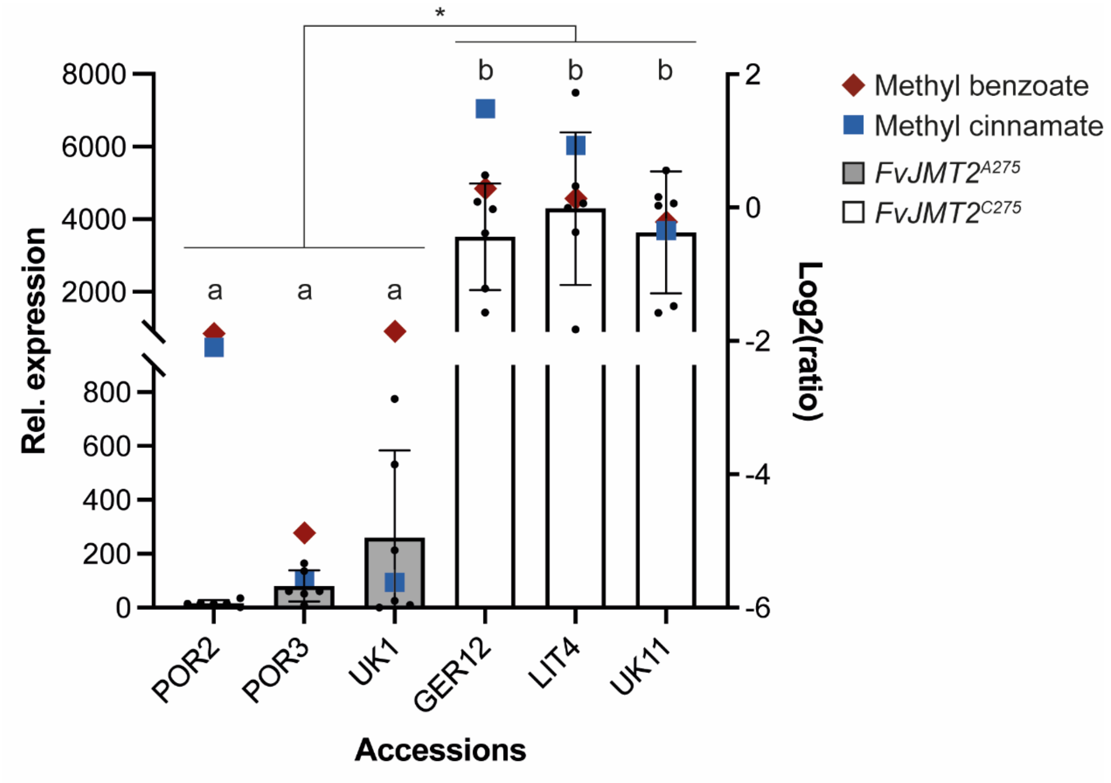

Figure S3. Relative *FvJMT2* expression and relative content of methyl benzoate and methyl cinnamate in *F. vesca* accessions carrying different allelic variants at SNP SFVB2_28340942. Accessions carrying the truncated *FvJMT2^{A275}* allele (POR2, POR3 and UK1; grey bars) are compared with accessions carrying the full-length *FvJMT2^{C275}* allele (GER12, LIT4 and UK11; White bars). Different letters indicate statistically significant differences in *FvJMT2* expression as determined by one-way ANOVA followed by Tukey's multiple comparations test ($p \leq 0.001$). Relative accumulation of methyl benzoate and methyl cinnamate in ripe fruit receptacles harvested is shown by diamonds and squares, respectively. Volatile compound data represent estimated marginal means derived from the combined dataset of the 2016 and 2017 harvest. Metabolite levels are expressed as the log2 ratios relative to a homogeneous control mixture composed of all analyzed samples. Asterisks indicate statistically significant differences in methyl benzoate and methyl cinnamate content between accessions carrying the A275 and C275 alleles, as determined by the Mann-Whitney U test performed independently for each compound ($p \leq 0.001$).

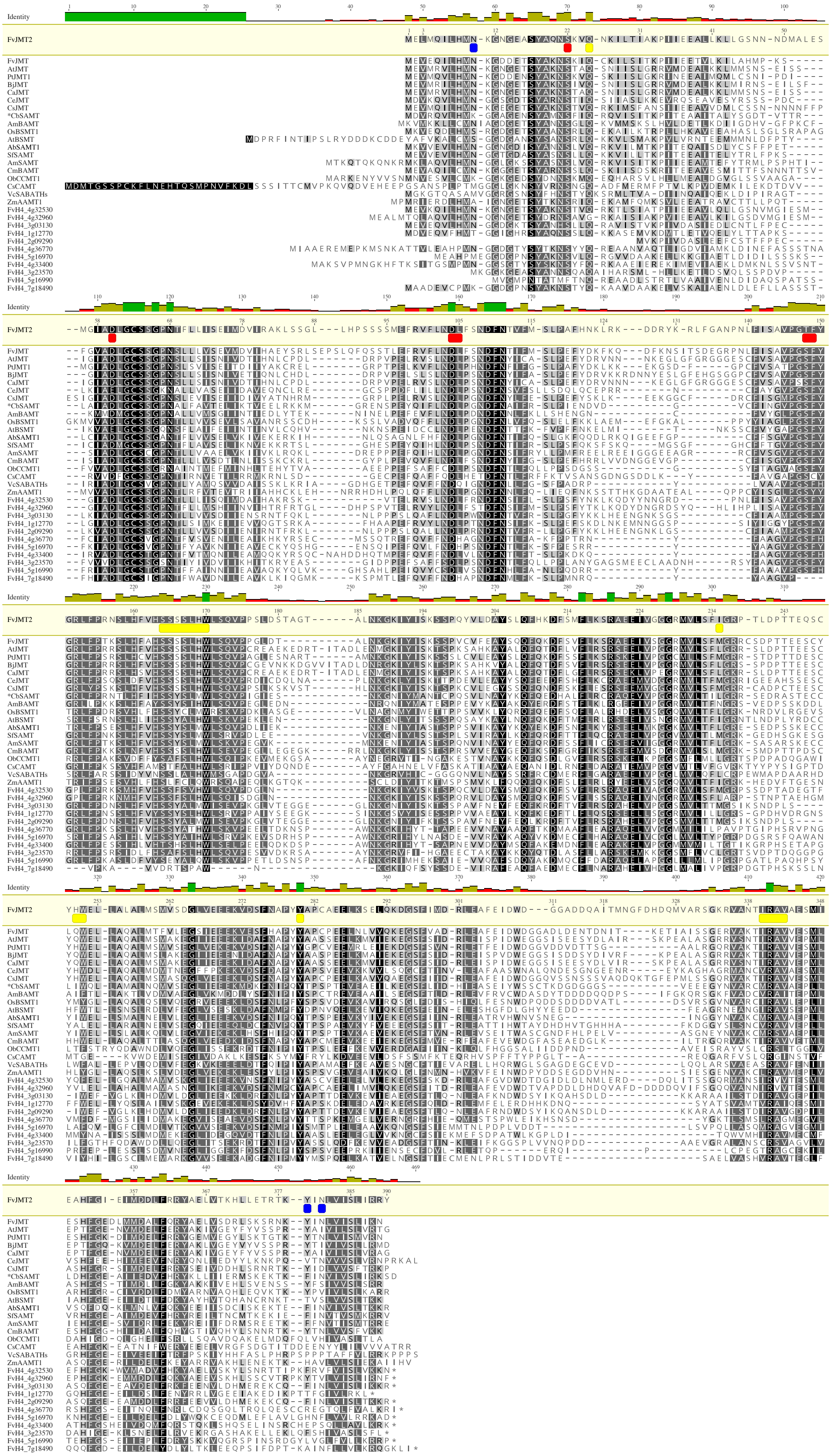
Identity
FvJMT2
FvJMT
AtJMT
PtJMT1
BjJMT
CaJMT
CeJMT
CsJMT
*CbSAMT
AmBAMT
OsBSMT1
AtBSMT
AbSAMT1
SfSAMT
AmSAMT
CmBAMT
ObCCMT1
CsCAMT
VcSABATHs
ZmAAMT1
FvH4_4g32530
FvH4_4g32960
FvH4_3g03130
FvH4_1g12770
FvH4_2g09290
FvH4_4g36770
FvH4_5g16970
FvH4_4g33400
FvH4_3g23570
FvH4_5g16990
FvH4_7g18490

Figure S4. Multiple protein sequence alignment of FvJMT2 with other functionally characterized SABATH enzymes. Conserved amino acid residues corresponding to SAM/SAH (red), substrate-binding sites (yellow), and additional active sites (blue) are highlighted according to the enzymatic structure of CbSAMT (*) resolved by Zubieta et al. [64]. Protein sequences were retrieved from GenBank (when available) or the corresponding publications: *Fragaria vesca* FvJMT [35], *Populus trichocarpa* PtJMT1 (KC894590), *Camellia sinensis* CsJMT (XP028087949), *Arabidopsis thaliana* AtJMT (AAG23343.1), *Brassica juncea* BjJMT (AY667499), *Capsicum annuum* CaJMT (DQ222856), *Cymbidium ensifolium* CeJMT (JQ360571), *Clarkia breweri* CbSAMT [34], *Atropa belladonna* AbSAMT1 (AB049752), *Stephanotis floribunda* SfSAMT (AJ308570), *Antirrhinum majus* AmSAMT (AF515284), *Cucumis melo* CmBAMT (XM_008468313.3), *Antirrhinum majus* AmBAMT (Q9FYZ9), *Arabidopsis thaliana* AtBSMT (BT022049*), Oryza sativa* OsBSMT1 (XM467504), *Ocimum basilicum* ObCCMT1 (EU033968), *Victoria cruziana* VcSABATHs (MZ541994), and *Zea mays* ZmAAMT (ZmAAMT1). *F. vesca* homologs were retrieved from the reference genome annotation v4.0a2 [49]. Amino acid residues are color-coded based on their similarity to the FvJMT2 reference sequence: black = 100%, dark grey = 80–100%, light grey = 60–80%, and white < 60% similarity.

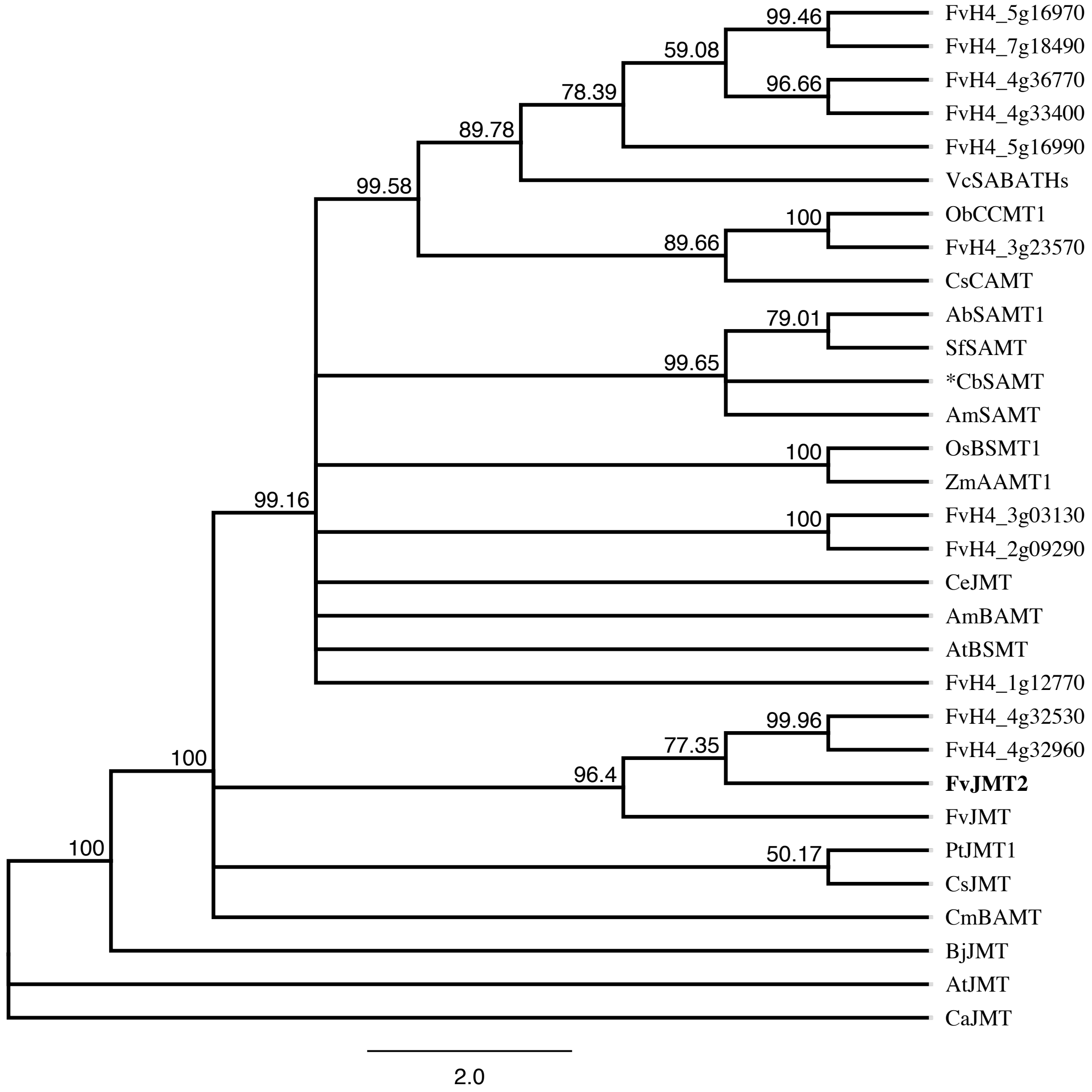

Figure S5. Neighbor joining consensus tree showing the relationship of FvJMT2 with SABATH enzymes from *F. vesca* and other plant species. *F. vesca* proteins correspond to homologous JMTs. The asterisk denotes the protein for which the crystal structure has been resolved (*Clarkia breweri* Salicylic Acid Carboxyl Methyltransferase (CbSAMT)) [34].

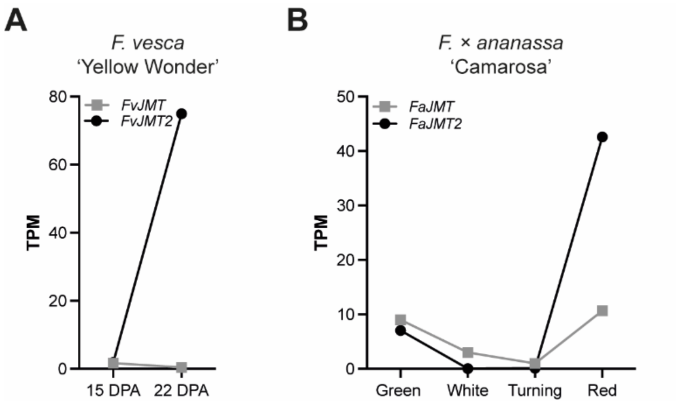

Figure S6. *JMT* and *JMT2* expression patterns during fruit ripening in both diploid and octoploid genome backgrounds. **A)** Expression of *FvJMT2* and *FvJMT* at two timepoints (15 and 22 days post-anthesis, DPA) during fruit ripening in *F. vesca* 'Yellow Wonder 5AF7', as reported by Li et al. [49] **B)** Expression of the *F. × ananassa* ortholog *FaJMT2* (FxaC_8g06390) and *FaJMT* (FxaC_7g41871) in the fruit receptacles at four developmental stages: immature green (Green, ~14 DPA), mature white (White, ~21 DPA), mature breaking (Turning, ~28 DPA), and mature ripe (Red, ~35 DPA), as previously reported [67,68].

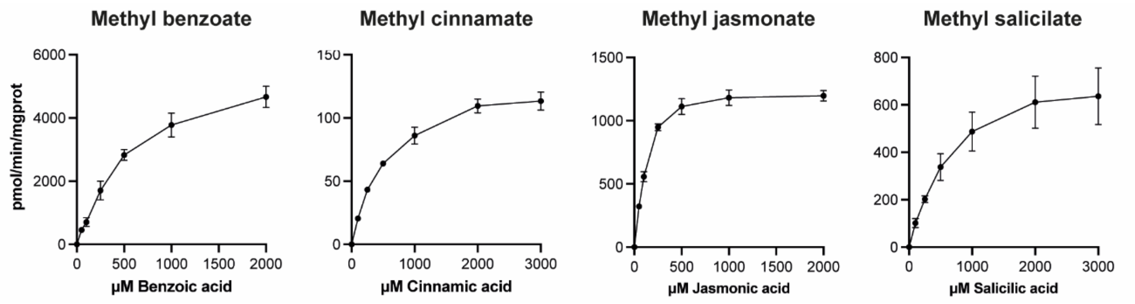

Figure S7. *In vitro* enzymatic kinetics of FvJMT2^{C275} with four major substrates. Michaelis-Menten curves showing the enzymatic activity of FvJMT2^{C275} in the presence of increasing concentrations of benzoic acid, cinnamic acid, jasmonic acid, and salicylic acid. Data represent the mean values of three independent assay replicates.
